# Potential of Bacterial Cellulose Chemisorbed with Anti-Metabolites, 3-Bromopyruvate or Sertraline, to Fight against *Helicobacter pylori* Lawn Biofilm

**DOI:** 10.3390/ijms21249507

**Published:** 2020-12-14

**Authors:** Paweł Krzyżek, Grażyna Gościniak, Karol Fijałkowski, Paweł Migdał, Mariusz Dziadas, Artur Owczarek, Joanna Czajkowska, Olga Aniołek, Adam Junka

**Affiliations:** 1 Department of Microbiology, Faculty of Medicine, Wroclaw Medical University, 50-368 Wroclaw, Poland; grazyna.gosciniak@umed.wroc.pl; 2 Department of Immunology, Microbiology and Physiological Chemistry, Faculty of Biotechnology and Animal Husbandry, West Pomeranian University of Technology in Szczecin, 70-311 Szczecin, Poland; karol.fijalkowski@zut.edu.pl; 3 Department of Environment, Hygiene and Animal Welfare, Wroclaw University of Environmental and Life Sciences, 51-630 Wroclaw, Poland; pawel.migdal@upwr.edu.pl; 4 Faculty of Chemistry, University of Wroclaw, 50-353 Wroclaw, Poland; mariuszdziadas@gmail.com; 5 Department of Drug Form Technology, Wroclaw Medical University, 50-556 Wroclaw, Poland; artur.owczarek@umed.wroc.pl; 6 Laboratory of Microbiology, Polish Center for Technology Development PORT, 54-066 Wroclaw, Poland; czajkowskaj@hotmail.com; 7 Faculty of Medicine, Lazarski University, 02-662 Warsaw, Poland; olga.aniolek@lazarski.pl; 8 Department of Pharmaceutical Microbiology and Parasitology, Wroclaw Medical University, 50-556 Wroclaw, Poland; feliks.junka@gmail.com

**Keywords:** *Helicobacter pylori*, bacterial cellulose, anti-biofilm activity, morphology, 3-bromopyruvate, sertraline

## Abstract

*Helicobacter pylori* is a bacterium known mainly of its ability to cause persistent inflammations of the human stomach, resulting in peptic ulcer diseases and gastric cancers. Continuous exposure of this bacterium to antibiotics has resulted in high detection of multidrug-resistant strains and difficulties in obtaining a therapeutic effect. The purpose of the present study was to determine the usability of bacterial cellulose (BC) chemisorbed with 3-bromopyruvate (3-BP) or sertraline (SER) to act against lawn *H. pylori* biofilms. The characterization of BC carriers was made using a N2 adsorption/desorption analysis, tensile strength test, and scanning electron microscopy (SEM) observations. Determination of an antimicrobial activity was performed using a modified disk-diffusion method and a self-designed method of testing antibacterial activity against biofilm microbial forms. In addition, bacterial morphology was checked by SEM. It was found that BC disks were characterized by a high cross-linking and shear/stretch resistance. Growth inhibition zones for BC disks chemisorbed with 2 mg of SER or 3-BP were equal to 26.5–27.5 mm and 27–30 mm, respectively. The viability of lawn biofilm *H. pylori* cells after a 4-h incubation with 2 mg SER or 3-BP chemisorbed on BC disks was ≥4 log lower, suggesting their antibacterial effect. SEM observations showed a number of morphostructural changes in *H. pylori* cells exposed to these substances. Concluding, SER and 3-BP chemisorbed on BC carriers presented a promising antibacterial activity against biofilm *H. pylori* cells in in vitro conditions.

## 1. Introduction

*Helicobacter pylori* of Epsilonproteobacteria class is a Gram-negative bacterium known primarily of its ability to cause persistent inflammation of the human stomach, resulting in such pathological alterations as peptic ulcer diseases, gastric cancers, and mucosa-associated lymphoid tissue lymphomas [1,2]. *H. pylori* colonizes and survives in the harsh environment of stomach thanks to the number of adaptations, which include spiral shape, urease secretion, and adhesin-mediated adherence to mucosa [3]. The variety and maliciousness of *H. pylori* virulence factors was the reason why the latest Maastricht V guidelines recommend to eradicate this bacterium, even if disease symptoms have not yet appeared [4].

Currently, antibiotics still remain the only acceptable treatment option against *H. pylori*. Regrettably, continuous exposure of *H. pylori* to these antimicrobials has resulted in alarmingly high detection of multidrug-resistant (MDR) strains [5]. The issue of aforementioned pathogens’ antibiotic resistance is an important and current topic [6]. The prevalence of antibiotic-resistant strains is considered so high that WHO has placed *H. pylori* on the list of pathogens whose treatment requires the development of new eradication standards [7]. Due to a lack of new solutions, a bismuth quadruple therapy (BQT) is presently recommended as a first line of treatment [8]. This therapy has a satisfactory level of approximately 90% eradication [9,10,11]. For the currently recommended BQT, however, a negative effect on patients’ microbiota has been demonstrated [12,13,14,15]. Additionally, the systemic BQT, despite its relatively good tolerance, contributes to the occurrence of side effects in patients, which in consequence may affect non-adherence and therapeutic failures [16]. Taking into account above-mentioned disadvantages of BQT and high adaptability of *H. pylori*, an implementation of novel, efficient anti-*H. pylori* compounds is of paramount importance [6].

There are two promising compounds, namely 3-bromopyruvate (3-BP) and sertraline (SER), for which an antimicrobial activity against planktonic and biofilm forms of different pathogens was demonstrated [17,18,19,20,21]. Both of them affect a number of targets in microbial cells, including protein synthesis, metabolic pathways, and production of intracellular ATP. Therefore, these substances are often referred to as anti-metabolites. Our team has already demonstrated not only the antibacterial activity of 3-BP and SER against planktonic *H. pylori* cells, but also synergistic/additive interactions of these compounds with antibiotics, providing proof of the concept for their future clinical application as adjuvants of these antimicrobials [22,23]. Despite the fact that pharmacological parameters and the level of cytotoxicity make the use of 3-BP and SER in a systemic therapy possible [24,25], this route of drug administration is frequently associated with such disadvantages as low bioavailability, limited penetration into affected tissues, and an induction of resistance among human microbiome representatives [26].

We hypothesize that 3-BP and SER should be provided to gastric mucosa, disturbed by the ongoing *H. pylori* colonization/infection, by a local administration. Such approach requires coupling of antimicrobial substances with an appropriate carrier, in order to provide them to the infection site. There is a number of carriers which could be possibly used for the delivery of these compounds, including liposomes, microspheres, nanoparticles, and a variety of polymeric carriers of artificial or natural origin [26,27,28]. In our opinion, bacterial cellulose (BC) produced by non-pathogenic *Komagataeibacter xylinus* is the most suitable among them, due to broad spectrum of favorable physical and chemical properties [26,27,28]. Compared to plant cellulose, BC of microbial origin has a higher degree of crystallization and polymerization, which translates into a higher absorption capacity. Therefore, it displays a very high ability to absorb and release drugs from within its porous structure. Moreover, it exhibits a high mechanical strength (resistance to shear) and a high elastic modulus (an ability to retain shape after stretching) [29]. Moreover, BC’s chemical composition is identical with composition of plant cellulose and is not digested in harsh environment of stomach [30]. Our team has already shown the suitability of BC chemisorbed with antibiotics and antiseptics to fight against biofilms formed by pathogens responsible for infections of wounds [31], bones [32,33,34], and oral cavity [35]. Therefore, the purpose of the present study was to determine the usability of BC chemisorbed with 3-BP and SER to act against biofilm of *H. pylori*.

## 2. Materials and Methods

### 2.1. Culture Conditions and Bacterial Strains Used

The research was carried out using two selected *H. pylori* strains, i.e., antibiotic-sensitive, reference Tx30a (ATCC 51932) stain from the American Type Culture Collection (ATCC) and triple-resistant, clinical 8064 strain (resistant to clarithromycin, metronidazole, and levofloxacin) belonging to the collection of the Department of Microbiology, Wroclaw Medical University. An antibiotic resistance profile has been established using E-tests and interpreted according to EUCAST (2019) recommendations [36]. Strains of *H. pylori* were kept at −70 °C in Tryptic Soy Broth (TSB; Oxoid, Dardilly, France) and 15% glycerol [22,23]. After thawing, bacteria were cultured on Columbia agars (Difco, Lublin, Poland) with 7% hemolyzed horse blood (CA+HB). Plates with seeded bacteria were directed to a 3-day incubation under microaerophilic conditions (Genbox microaer kits, BioMerieux, Marcy-l’Étoile, France) at 37 °C.

### 2.2. Carrier Characteristics

In order to obtain BC membranes of the same diameter, stationary culture of *Komagataeibacter xylinus* DSM 46602 were grown in the Herstin–Schramm medium in a 24-well plate (Nest Biotechnology Co., Wuxi, China) for 7 days at 28 °C. The BC pellicles harvested from the medium were next purified by alkaline lysis, washed with distilled water, autoclaved, and kept in 4 °C for the time of further analyses.

To determine the wet and dry weight of the samples, BC pellicles were weighed using an analytical balance and then dried at 60 °C overnight and weighed again (WTB 2000 Radwag, Radom, Poland). The microstructure of the BC membrane was analyzed using SEM (Auriga 60, Zeiss, Jena, Germany). BC membranes were fixed in glutaraldehyde (POCH, Gliwice, Poland) and subjected to the sputtering with Au/Pd (60:40) using a high vacuum coater (EM ACE600, Leicasputter, Leica Microsystems, Wetzlar, Germany). The porosity of BC surface was analyzed by means of ImageJ software (NIH, Bethesda, Rockville, MA, USA). The N_2_ adsorption/desorption isotherms at 77 K were measured using a Micromeritics ASAP 2010 M instrument (Micromeritics, Norcross, GA, USA) and the specific surface area was calculated by the Brunauer–Emmett–Teller method. The pore volume and pore diameter of whole sample were calculated by the Barrett–Joyner–Halenda method. The BC dressings’ tensile strength test was performed using an MTS Synergie 100^®^ machine (MTS System Corp, Eden Praire, MN, USA). The tests were carried out at a speed of 10 mm/min at room temperature. Based on the recorded values of force and displacement, stress–strain graphs were prepared; and based on these graphs, mechanical parameters (such as tensile strength) were determined. Moreover, to determine an impact of low pH on BC, samples were introduced to 5 M and 10 M HCl (Poch, Wrocław, Polska) for 4 h. The BC incubated in 0.9% NaCl solution for 4 h served as control samples. After incubation, samples were taken out of solutions and analyzed visually with regard to their shape and color.

Additionally, the release rate of two tested substances (SER and 3-BP) using spectrometry was also determined. SER-chemisorbed BC carriers were placed in glass vessels containing 10 mL of a mixture methanol:water (50:50 *v/v*) serving as the drug release medium. The absorbance of SER solutions was measured at 273 nm (Thermo Scientific Multiscan GO spectrometer). Analysis of 3-BP was performed on a model 1260 HPLC-UV/VIS system (Agilent, Santa Clara, CA, USA) on a Zorbax SB-C8 chromatography column, 80 × 4.6 mm, 5 µm (Grom, Germany). During this process a flow rate of 1 mL/min in isocratic conditions using water:acetonitrile:phosphoric acid (9:1:0.1 *v/v*) was applied. The results were integrated and analyzed with the Chemstation B.04 software (Agilent, USA).

### 2.3. Determination of Antimicrobial Activity of Tested Compounds

A microdilution method in 12-well titration plates (Bionovo, Legnica, Poland) was used to estimate minimal inhibitory concentrations (MICs) and minimal bactericidal concentrations (MBCs) for the two tested *H. pylori* strains [22,23]. The 1 mL of suspension was prepared in each well by diluting tenfold an initial bacterial suspension (10^8^ CFU/mL, 4 McFarland units) in Brain Heart Infusion broth (BHI; Oxoid, Dardilly, France) with 7% foetal calf serum (FCS; Gibco, Paisley, Scotland, UK) (BHI+FCS), thus resulting in the density of 10^7^ CFU/mL. In addition, each well contained a concentration gradient of one of the tested compounds, i.e., SER (0.25–16 µg/mL, Sigma-Aldrich, St. Louis, MO, USA), 3-BP (8–512 µg/mL, Sigma-Aldrich), bismuth subsalicylate (BIS, 0.5–32 µg/mL, Sigma-Aldrich), and amoxicillin (AMX, 0.015–0.96 µg/mL, Sigma-Aldrich). Subsequently, plates containing bacteria were incubated at 37 °C per 3 days in microaerophilic conditions and provided rotation of 100 rpm (MaxQ 6000, ThermoFisher, Waltham, MA, USA).

The activity of SER or 3-BP released from BC carriers was determined by a modified diffusion-disk method as previously described [33,37]. The paper disks chemisorbed with the tested compounds were applied as a control setting of the experiment. In the case of AMX, ready-made paper disks were used (25 µg/mL Oxoid, France). The tested compounds were initially dissolved in DMSO (Sigma-Aldrich) and then diluted in BHI broths to obtain the final DMSO concentration ≤1%. The paper and BC disks chemisorbed with BIS or AMX and 1% DMSO solution served as a positive and negative controls of experiments, respectively. One centrally located cellulose disk (15 mm) or three evenly spaced paper disks (6 mm) were placed on CA+HB plates containing a lawn of bacteria (approx. 5 × 10^6^ CFU/mL). Culture media with sown bacteria and with applied disks (0.2, 1 and 2 mg for SER, 3-BP and BIS, or 25 µg for AMX per disk) were incubated under microaerophilic conditions at 37 °C for 3 days. Additionally, the morphology of antimicrobial-treated *H. pylori* was determined by directing agar fragments from the area with a bacterial lawn (a positive control) and inhibited growth for a scanning electron microscopy (SEM) imaging.

**Figure 1:**
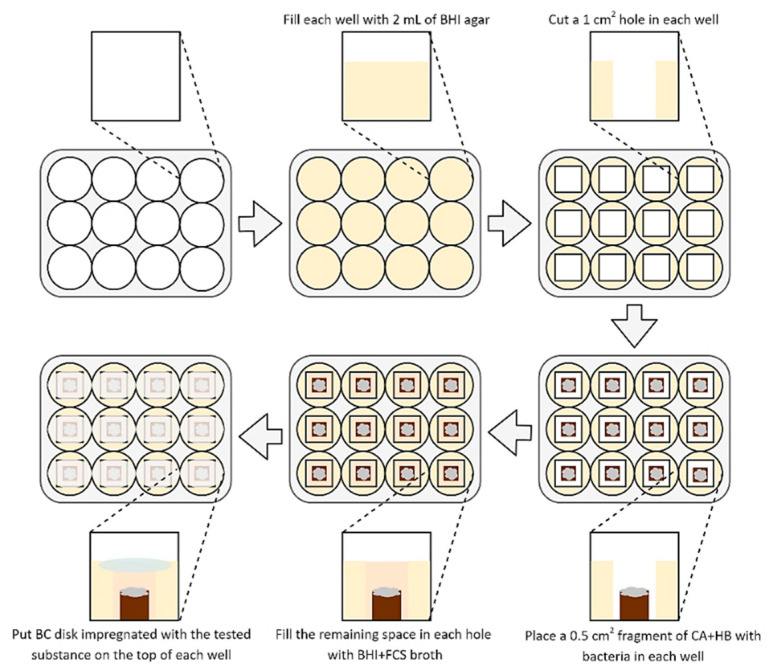
Diagram presenting a method used to determine an antimicrobial activity of tested substances released from BC carriers against 3-day lawn biofilm *H. pylori* cells. Abbreviations: BHI agar, Brain Heart Infusion agar; BHI+FCS broth, Brain Heart Infusion broth with 7% foetal calf serum; CA+HB, Columbia agar with 7% hemolysed horse blood.

To determine the activity of BC disks impregnated with the tested substances, a methodology established by Krasowski and Junka et al. (2019) [35] was used (Figure 1). The 2 mL of BHI agar (Oxoid, France) was added into each well of a 12-well titration plate and allowed to solidify for a one day. Then, 10 mm^2^ agar fragments were cut out from each well. Following this, 5 mm^2^ fragments of CA+HB agar with a three-day lawn *H. pylori* biofilms were placed in the empty space of each well (the lawn biofilms growth was done according to Dusane et al. (2019) and Lochab et al. (2020) [38,39], with an incubation at 37 °C and microaerophilic atmosphere). After this stage, the wells were filled with approx. 0.25 mL of BHI+FCS to obtain a convex meniscus. Finally, each well was covered from above with a BC disk containing 0.2, 1, or 2 mg of the tested compounds (SER or 3-BP). The positive and negative control were disks with BIS or AMX and disks non-impregnated with any substance, respectively. Such bacteria were then cultured for 1–4 h at 37 °C, microaerophilic conditions, and 100 rpm shaking.

The viability of *H. pylori* strains was determined by plating antimicrobial-treated bacteria and counting an amount of grown colonies. Agar fragments containing bacteria were mechanically crushed in 1 mL of BHI+FCS (homogenization according to Cooke et al. (2019) [40]). The resulting suspension was then serially diluted (10^1^–10^3^) and 20 µL of fluid was plated on CA+HB agars. Such plates were incubated for 3 and 7 days under microaerophilic conditions and at 37 °C, and the number of grown colonies was counted independently for each time point. The viability was confirmed independently by using BacLight Live/Dead staining (L7012, ThermoFisher, USA) and fluorescence microscopy (Olympus BX51, Japan). Using the ImageJ program, determination of the green/red fluorescence ratio was made in relation to five regions of interests from three photos obtained from separate tests [22,23]. The morphology of antimicrobial-treated bacteria was determined by directing the agar fragments with bacteria for SEM [22,23]. The agar fragments were immersed in 0.2 mL of 2.5% glutaraldehyde (Sigma-Aldrich), washed two times with phosphate buffer (Sigma-Aldrich), and passed through an ethanol series. Such samples were dried at room temperature, sprayed with 15 nm of gold, and observed using SEM (Auriga 60, Zeiss, Germany).

### 2.4. Statistical Analysis

Statistical differences were assessed using the Kruskal-Wallis test with a post-hoc Dunnett’s analysis. Data is presented as the means ± standard errors of the means (SEM) obtained from three different measurements (+technical repeats). All analyses were considered statistically significant when the *p* value was less than 0.05.

## 3. Results

In the first line of experiments, we applied a standard titration plate assessment of MICs and MBCs to confirm whether 3-BP and SER display an antimicrobial activity against the tested *H. pylori* strains. In the same setting, also BIS and AMX were used as substances of already confirmed activity against *H. pylori*. The highest antibacterial activity was demonstrated for AMX with MICs ranging from 0.06–0.12 µg/mL. We found that the multidrug resistant *H. pylori* 8064 (resistant to clarithromycin, metronidazole, and levofloxacin) was more sensitive to AMX (MIC = 0.06 µg/mL and MBC = 0.12 µg/mL) than the antibiotic-sensitive *H. pylori* Tx30a (MIC = 0.12 µg/mL and MBC = 0.48 µg/mL). This is consistent with the observations of others showing that antibiotic resistance in *H. pylori* is associated with point mutations in drugs’ targets and their presence results in insensitivity to a given group of antibiotics, but does not translate into resistance to other antibiotics [41,42]. Additionally, it is worth mentioning that with regard to the antimicrobial activity, SER had relatively comparable properties to BIS (2–4 µg/mL and 2 µg/mL, respectively) (Table 1). The 3-BP, although also able to act against *H. pylori*, had MIC at the level of a dozen times higher than the two substances mentioned earlier (128 µg/mL) (Table 1).

**Figure 2:**
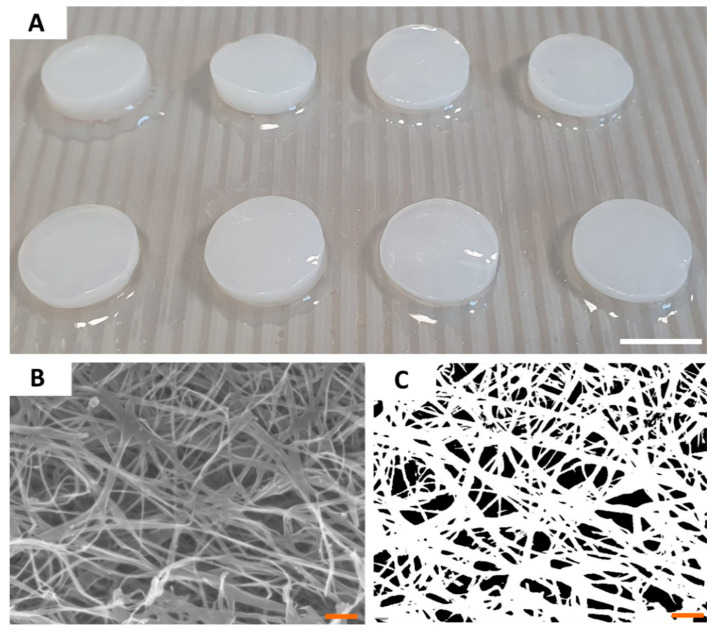
The macroscopic (**A**) and microscopic (**B**,**C**) pictures of BC carriers. The picture (**B**) was taken using SEM Auriga 60 microscope under magnification equal 50,000×; and subjected to re-processing (**C**) which allowed to calculate the porosity of carrier surface. Scale bars are equal to 1 cm in photo (**A**) and 1 µm in photos (**B**,**C**).

Having the ability of the tested compounds to act against *H. pylori* proven, we characterized the most crucial properties of BC as drug carriers. Because all BC carriers were cultured and purified within single production process, their parameters were of high consistency and comparability between particular disks. The diameter of BC carriers was 1.5 cm and its average wet and dry weight was equal to 0.5 g and 0.004 g, respectively. SEM analysis of a BC microstructure revealed pore-forming 3-D network of intertwined cellulose fibers (Figure 2). By means of a post-SEM image processing, the porosity of BC surface was established on 92 ± 126 nm, averagely. Using N2 adsorption/desorption analysis, we showed that BC carriers displayed average surface area of 9.37 ± 0.28 m^2^/g, average pore volume of 0.026 ± 0.007 cm^3^/g, and average pore diameter of 3.01 ± 0.18 nm. Another key parameter of a carrier to be applied in the harsh environment of stomach is mechanical strength (resistance to shear). Analysis we performed revealed that obtained BC carriers displayed the average tensile strength of 4.27 ± 0.77 MPa and average Young’s modulus equal 11.79 ± 1.05 MPa. Moreover, BC was resistant to digestion in in highly acidic conditions (pH < 2) during 4 h of incubation (Supplementary Figure S1).

**Figure 3:**
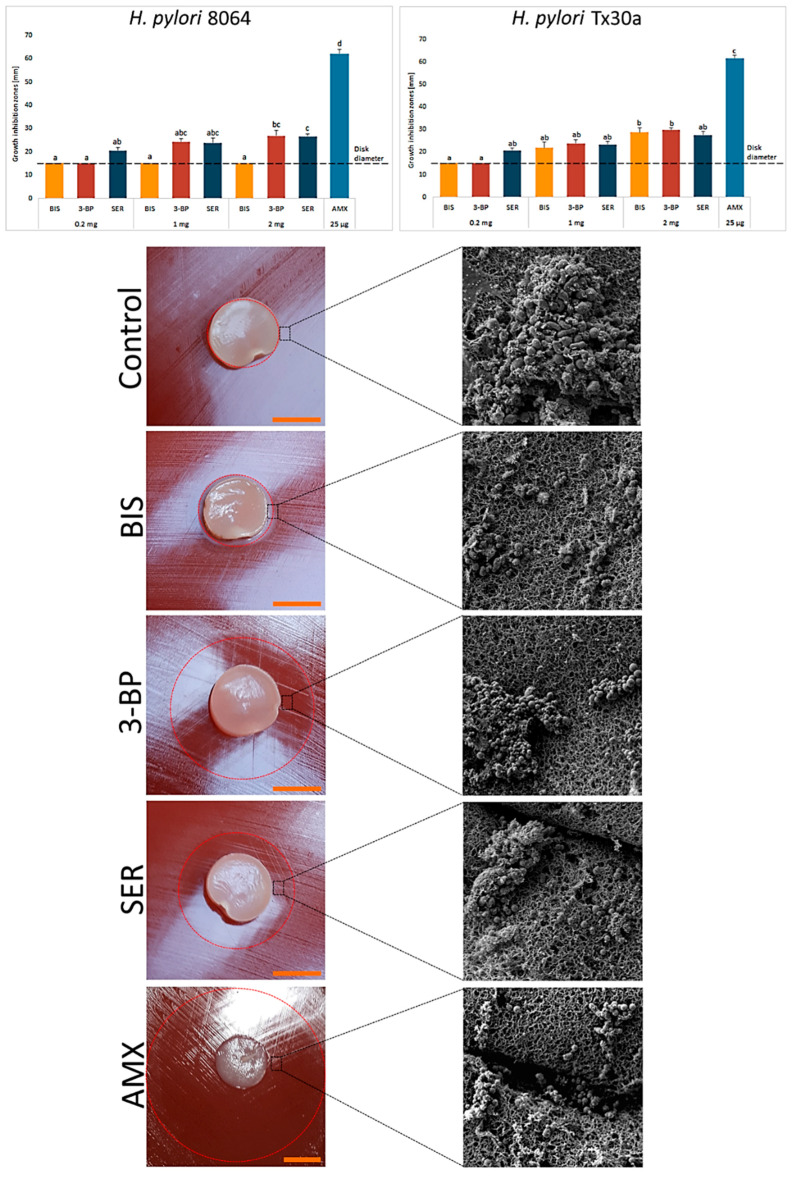
Activity of bismuth subsalicylate (BIS), 3-bromopyruvate (3-BP), sertraline (SER), and amoxicillin (AMX) released from BC carriers against *H. pylori* 8064 and Tx30a strains measured by a modified disk-diffusion method. Asterisks stand for a statistical significance (K-W test with post-hoc Dunn’s analysis). The dot-line at the value of 15 mm represents the diameter of BC carrier. Representative photos of *H. pylori* 8064 growth inhibition zones and cell morphology after exposure to BC carriers not chemisorbed (a negative control) or the tested compounds. Columns with the same subscript letters (a, b, c) are not significantly different from each other (*p* > 0.05). The presented results are the average of three independent biological tests (*n* = 3). Scale bar is equal to 1 cm.

After the stage of characterization of BC carriers, we chemisorbed them with the tested compounds. Using spectrometry, we observed that after 1 h ~31% and ~42% of SER and 3-BP, respectively, was released from BC disks. A prolonged incubation did not increase significantly the amount of resorbed compounds (Supplementary Figures S2 and S3). Obtained results confirmed the ability of BC carriers to chemisorb SER and 3-BP. Therefore, in our next line of investigation we analyzed their activity against *H. pylori* strains using a modified disk-diffusion method. As it can be seen in Figure 3, the novel 3-BP and SER compounds displayed equal (e.g., 1 mg and 2 mg for *H. pylori* Tx30a, *p* > 0.05) or higher (2 mg for *H. pylori* 8064, *p* < 0.05) activity against the tested *H. pylori* strains in comparison to BIS. Among the tested compounds, even despite its low dose of 25 µg, AMX had the highest activity (*p* < 0.05). In addition, it was noticed that unlike non-treated bacteria (a bacterial lawn area) being a mixed population of spiral and coccoid forms, the cells in zones of inhibited growth were only in the spherical form (Figure 3). The data concerning control settings, where paper disks chemisorbed with the tested substances, were applied as presented in Supplementary Figure S4.

**Figure 4:**
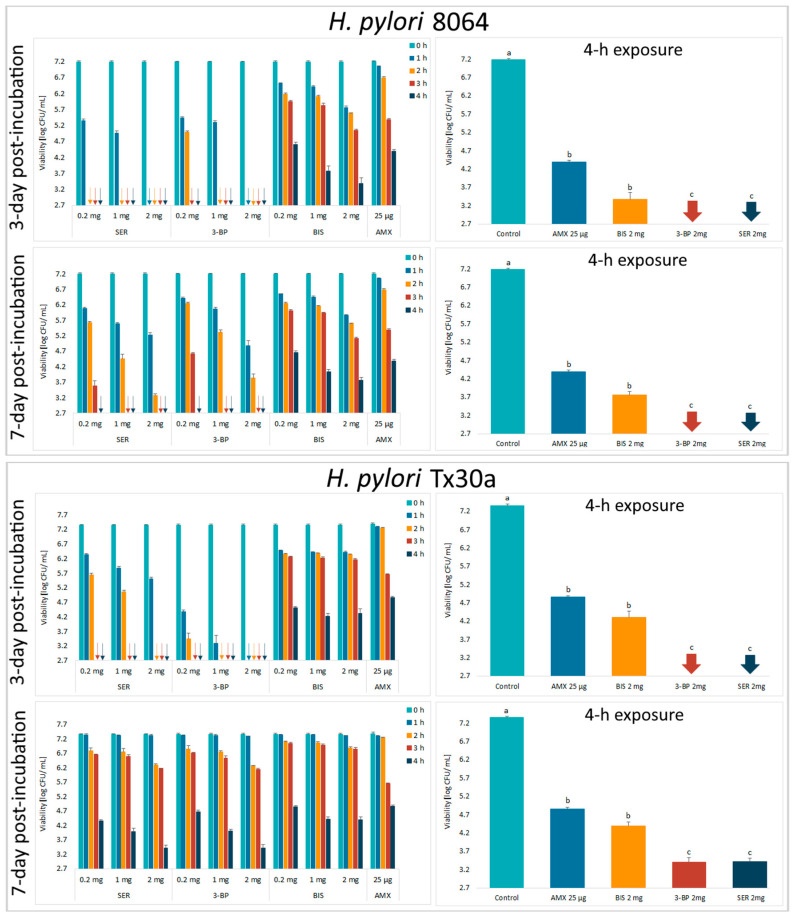
Viability of lawn biofilm of *H. pylori* 8064 and Tx30a strains after treatment for 1 h, 2 h, 3 h, and 4 h with BC carriers chemisorbed with bismuth subsalicylate (BIS); 3-bromopyruvate (3-BP), sertraline (SER), and amoxicillin (AMX). The colony forming units (CFUs) counting was performed after 3 or 7 days of culturing after exposure to these antimicrobials (the post-treatment period). The arrows indicate values below the detection threshold (500 CFU/mL, log_10_ = 2.7). Columns with the same subscript letters (a, b, c) are not significantly different from each other (*p* > 0.05). The presented results are the average of three independent biological tests (*n* = 3). The results of comparing the statistical significance of all tested samples are presented in the Figure S5.

Next, the activity of tested compounds was assessed by a self-designed method of testing antibacterial activity against lawn biofilms (Krasowski, Junka et al., 2019) [35] (Figure 1). In this experimental model, it was observed that survival of the *H. pylori* strains was negatively correlated with the time of exposure to BC chemisorbed with antimicrobials and their concentrations (Figure 4 and Supplementary Figure S4). Moreover, it was discovered that SER and 3-BP showed a higher activity against 3-day-old lawn *H. pylori* biofilms than BIS or AMX (*p* < 0.05). It is worth to notice that in comparison to the control samples, a 4-h incubation with all tested compounds decreased the viability of lawn *H. pylori* biofilms significantly (*p* < 0.05).

**Figure 5:**
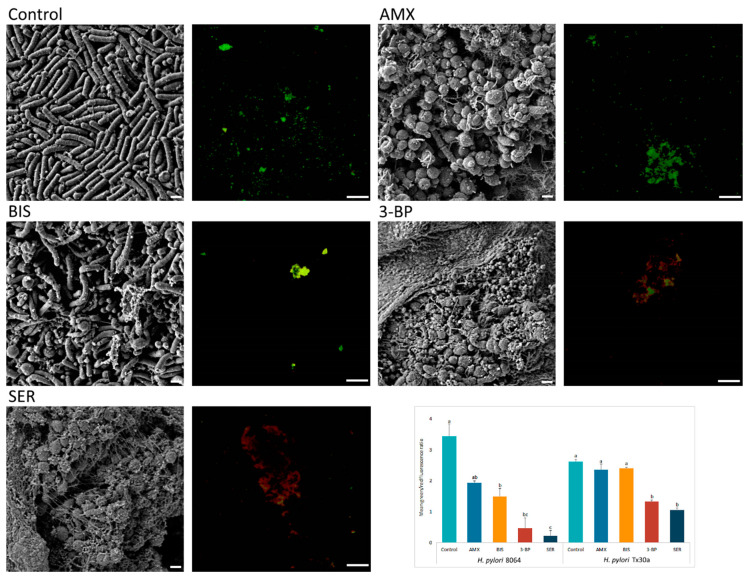
Representative scanning electron and fluorescence microscopy images showing an antibacterial activity of tested compounds (bismuth subsalicylate (BIS), 3-bromopyruvate (3-BP), sertraline (SER), and amoxicillin (AMX)) released from BC carriers after 4-h exposure against 3 day-old, lawn biofilm *H. pylori* cells. The small spherical structures (50–300 nm) are outer membrane vesicles (OMVs) secreted by bacteria. Green dye (SYTO 9) indicates live cells, while red (propidium iodide) indicates dead cells. The graph shows the ratio of mean green/red fluorescence of lawn biofilm *H. pylori* cells treated for 4 h with the tested substances. Columns with the same subscript letters (a, b, c) are not significantly different from each other (*p* > 0.05), counted separately for each strain. The presented results are the average of three independent biological tests (*n* = 3). Scale bar for SEM and fluorescence microscopy is 2 μm and 20 μm, respectively.

Above-presented results were additionally confirmed by microscopy. Fluorescence microscopy showed a decrease in the amount of live, biofilm *H. pylori* cells upon exposure to 3-BP and SER released from BC carriers (*p* < 0.05). For example, compared with untreated cells mean green/red fluorescence was 7- and 15-fold lower in biofilm *H. pylori* cells exposed to 3-BP and SER, respectively. Surprisingly, exposure of these bacteria to AMX had a low impact on the viability of biofilm cells (*p* > 0.05, Figure 5). To broaden the picture of the phenomenon, the analysis was extended by SEM (Figure 5). It was observed that *H. pylori*, placed on solid agars and immersed in liquid medium, formed highly complex aggregates consisted of numerous rod-shape cells (a positive control). Moreover, an existence of single small outer membrane vesicles (mostly OMVs of approx. 50 nm) was observed. After exposure on BIS, *H. pylori* still maintained mainly its rod-like morphology, although some cells transformed into coccoid forms. Cells of *H. pylori* treated with SER, 3-BP or AMX were present in the spherical morphotype only. Furthermore, numerous OMVs covering bacteria (mainly with 100–300 nm diameters) and the existence of intercellular junctions taking the shape of short or very long fibrils (particularly intensely visible for SER-treated cells) were noticed.

## 4. Discussion 

The conception of research presented in this article is based on a scientific re-perception approach [43], developed systematically by our team, especially with regard to bacterial cellulose [32,33,34]. Re-perception relies basically on application of specific material or device in new environment to obtain favorable and often unexpected outcomes. An example of re-perception approach is use of BC as a carrier of microorganisms [44] or as a local drug-delivery carrier [45], with the second being applied in this work. There is a bulk of evidence showing that use of local drug-delivery systems (liposomes, microspheres, nanoparticles, or polymeric carriers) is an effective and safe type of antimicrobial therapies, which is of particular importance in combating infections caused by multidrug-resistant pathogens [24,25,26] and may also find a broad application in the treatment of *H. pylori* infections. 

Substances that may be helpful in fighting *H. pylori* infections are 3-BP and SER. In previous studies, our research group showed an antimicrobial activity and synergistic/additive interactions with antibiotics of both these compounds against planktonic *H. pylori* forms [22,23]. Even if the systemic use of 3-BP or SER in the treatment of *H. pylori* is possible [24,25], methods contributing to the reduction/elimination of negative effects associated with the *H. pylori* eradication are an important stream of future research [26]. Therefore, our aim was to couple 3-BP and SER with BC carriers to analyze an in vitro potential of this local drug-delivery system to be applied as a counter-measure against *H. pylori* biofilms.

In the first line of our investigation we checked whether analyzed SER or 3-BP alone (non-coupled with a BC carrier) displayed the desired antibacterial activity and compared received results with values obtained for BIS and AMX, both of which are substances of the clinical use (Table 1). It occurred that all tested compounds were able to inhibit or kill *H. pylori*. Whereas AMX had the highest activity (MIC = 0.06–0.12 µg/mL), 3-BP displayed unquestionably the lowest activity (MIC = 128 µg/mL). Despite unfavorable initial results obtained for 3-BP, we decided to include this substance in further stages of experiment taking into account fact that local drug-delivery carriers allow to provide high concentrations of compounds into selected area.

Therefore, next we produced BC carriers and characterized their properties. The whole batch of BC was produced in the single cultivation time interval and cleansed using alkaline lysis in a single container, thus allowing us to obtain carriers of exact/almost exact features. Thanks to this approach, we obtained BC carriers of desired shape, porosity, and other parameters (Figure 2), comparable to these presented in our earlier work [35]. Moreover, we showed that BC was not digested and did not alter its structure in the highly acidic environment resembling conditions in stomach (Supplementary Figure S1). The porosity of BC presented in Figure 2, is a BC key parameter with regard to an ability of this biomaterial to absorb fluids. Therefore, the immersion of BC carriers with SER or 3-BP resulted in the chemisorption of these compounds within cellulose matrix as it was proven by a spectroscopy.

The results of a preliminary, modified disk-diffusion test confirmed that analyzed compounds were chemisorbed within BC and also shown their effective release resulted in formation of growth inhibition zones of *H. pylori* (Figure 3). Results presented in Figure 3, concerning comparison of 3-BP and BIS activity, stay in contrast to these obtained by means of the standard microplate assay (Table 1). It explicitly highlights not only dependence between results obtained and the type of test applied, but also the obvious need of using various tests to fully understand phenomena related with an assessment of microbial sensitivity to drugs. Also the chemical nature of the substance correlates with its antimicrobial activity [46]. 3-BP is a low-molecular weight compound (166 g/mol), while molecular weight of bismuth subsalicylate (BIS) is more than two times higher (361 g/mol). A positive correlation between low-molecular weight of antimicrobials and their ability to penetrate the biofilm matrix has been already demonstrated by other research teams [47,48]. Therefore, it can be also assumed that low-weight 3BP would display significantly higher penetration through agar (a disk-diffusion assay) and biofilm matrix (an antimicrobial assay against lawn biofilms) compared to BIS.

Following this idea, we performed an additional, self-developed experimental setting [35,37], in which active compounds released from a BC carrier need to penetrate through liquid microbiological medium and then through 3 day-old, lawn biofilm *H. pylori* cells (Figure 1). It is worth noting that bacterial cells tested in the current experiments were adhered to the agar surface and immersed in the nutrient medium [38,39], while an alternative model focuses on biofilms and cell aggregates formed at the air-liquid surface [49]. Such procedure may affect the different physiology of *H. pylori* cells, while it guarantees obtaining high cell biomass, which is difficult to obtain for *H. pylori* using classical methods. The porosity of surface (enabled in the experimental setting by application of agar-based medium) is a known factor increasing adherence of settled communities of microbes [50] and it allows to obtain more repeatable results than in case when smooth polystyrene and polypropylene surfaces are used for in vitro testing [51]. The proper surface porosity correlates with a higher adherence of cells, which in turn decreases the effect of aggressive pipette-based washing, i.e., the random de-attachment of large amounts of biofilm and/or multicellular aggregates [52]. Therefore, the results obtained in the aforementioned experimental setting (with all limitations relevant to in vitro analyses) may serve as first and prerequisite proof of the concept, which could be developed later on in animal and clinical studies. 

Results presented in Figure 4 and Figure S5 revealed a direct time-dependent and dose-dependent antibacterial effect of these compounds against lawn *H. pylori* biofilms. For both strains, a growth inhibition effect was significantly higher when an amount of grown colonies was read after three days of incubation than after a one week of culture (the post-treatment period). The reason of this phenomenon is unknown, but it seems that this mechanism was associated with an ability of both 3-BP and SER to reduce metabolism and intracellular ATP level [19,20,21]. This difference was not seen in bacteria treated with BIS or AMX, although there are studies showing an ability of BIS to disrupt *H. pylori* metabolism [53,54,55]. It is possible that SER, 3-BP and BIS affect the metabolism of these bacteria, but may interfere with various metabolic pathways.

The impact of 3-BP and SER has been extended to assess morphological changes in *H. pylori*, a process for which an involvement in antibiotic tolerance is suggested [56,57]. Bacteria treated with these substances underwent morphological transition to spherical cells (Figure 3 and Figure 5). However, an analysis using fluorescence microscopy showed that the morphological transformation was not protective, because an exposure to these compounds was accompanied by a significant reduction in green fluorescence (Figure 5). The antibacterial nature of 3-BP and SER, as well as the lack of protective function of coccoid forms, are consistent with previous observations of our research group when testing 3-BP and SER against planktonic *H. pylori* forms [22,23]. It is worth noting that *H. pylori* cells treated with BIS or AMX maintained a high level of green fluorescence (Figure 5). This phenomenon seems to be particularly interesting in the case of AMX, because this antibiotic had a very high bactericidal activity against planktonic forms (a microdilution method, MIC = 0.06–0.12 µg/mL) and freshly seeded cells settled on the agar (growth inhibition zones produced by 25 µg/disk being ~60 mm), while significantly lower in relation to 3 day-old, lawn *H. pylori* biofilms (reduction of CFU/mL after a 4-h incubation was <3 logs). A high level of green fluorescence suggests that the observed decrease in CFU/mL is rather associated with the loss of culturability in the process of transformation into coccoid forms than the reduction of viability [57]. Our observations are consistent with the reports of others indicating a low bactericidal activity of AMX against biofilm *H. pylori* forms [58,59].

The SEM observation of *H. pylori* cells treated with 3-BP, SER, BIS, or AMX, in addition to morphological transformation, also revealed a presence of a high amount of OMVs. These structures were produced in a significantly higher amount compared to the control samples and were very often larger than in non-treated ones (Figure 5). Proteomic studies of *H. pylori* OMVs have shown that the size of these structures determines their content and protein composition, which in turn may translate into their functions [60,61]. The source of OMVs and an involvement of these structures in antibiotic tolerance of *H. pylori* exposed to antimicrobial substances is another interesting direction of research. We suggest that the presence of numerous OMVs in *H. pylori* biofilm may determine the low sensitivity of this bacterium to AMX and BIS. The OMVs produced by *H. pylori* possess numerous proteins [60,61] and extracellular DNA (eDNA) particles [62,63], both anchored to the surface of these organelles. The OMVs-dependent tolerance of biofilm *H. pylori* forms to AMX and BIS could be associated with the presence of beta-lactamases capable of breaking down AMX [61] or eDNA-related chelation of metal ions (including BIS) [64,65]. The suggested mechanism, however, should be experimentally verified.

## 5. Conclusions

In conclusion, the data presented in this article show that SER and 3-BP may be useful in the treatment of *H. pylori.* Additionally, coupling of these substances with BC carriers may be a promising approach directed to more accurate and efficient treatment of this pathogen. This trend will have to be verified in future in vivo studies.

## Figures and Tables

**Table 1:** MICs and MBCs of amoxicillin (AMX), bismuth subsalicylate (BIS), sertraline (SER), and 3-bromopyruvate (3-BP) against *H. pylori* Tx30a and 8064 strains.

*H. pylori* Strains	SER		3-BP		AMX		BIS	
	MIC	MBC	MIC	MBC	MIC	MBC	MIC	MBC
Tx30a	4	8	128	128	0.12	0.48	2	2
8064	2	2	128	128	0.06	0.12	2	4

MICs and MBCs are expressed as [µg/mL].

## Supplementary Materials

The following are available online at https://www.mdpi.com/1422-0067/21/24/9507/s1, Figure S1. Bacterial cellulose incubated in low pH does not alter its structure. Figure S2. Time-dependent release of 3-bromopyruvate (3-BP) from bacterial cellulose (BC) disks. Figure S3. Time-dependent release of sertraline (SER) from bacterial cellulose (BC) disks. Figure S4. Activity of tested compounds released from paper disks against *H. pylori* 8064 and Tx30a strains measured by a modified disk-diffusion method. Figure S5. Checkerboards showing statistically significant differences between the viability of lawn biofilm cells of *H. pylori* Tx30a and 8064 strains exposed to BC carriers chemisorbed amoxicillin (AMX), bismuth subsalicylate (BIS), sertraline (SER), and 3-bromopyruvate (3-BP) over time. The red and white fields represent a statistically significant (*p* < 0.05) and not significant (*p* > 0.05) differences, respectively (K-W test with a post-hoc Dunn’s analysis). Graphical presentation of the results made on the basis of Tripathy et al. (2020) [66].
