# Supplementary material for: Potential of Bacterial Cellulose Chemisorbed with Anti-Metabolites, 3-Bromopyruvate or Sertraline, to Fight against Helicobacter pylori Lawn Biofilm
Source: Int J Mol Sci. 2020 Dec 14;21(24):9507. doi: 10.3390/ijms21249507 (PMC7765062; doi:10.3390/ijms21249507)
Supplement: Supplementary file 1 [file ijms-21-09507-s001.pdf]

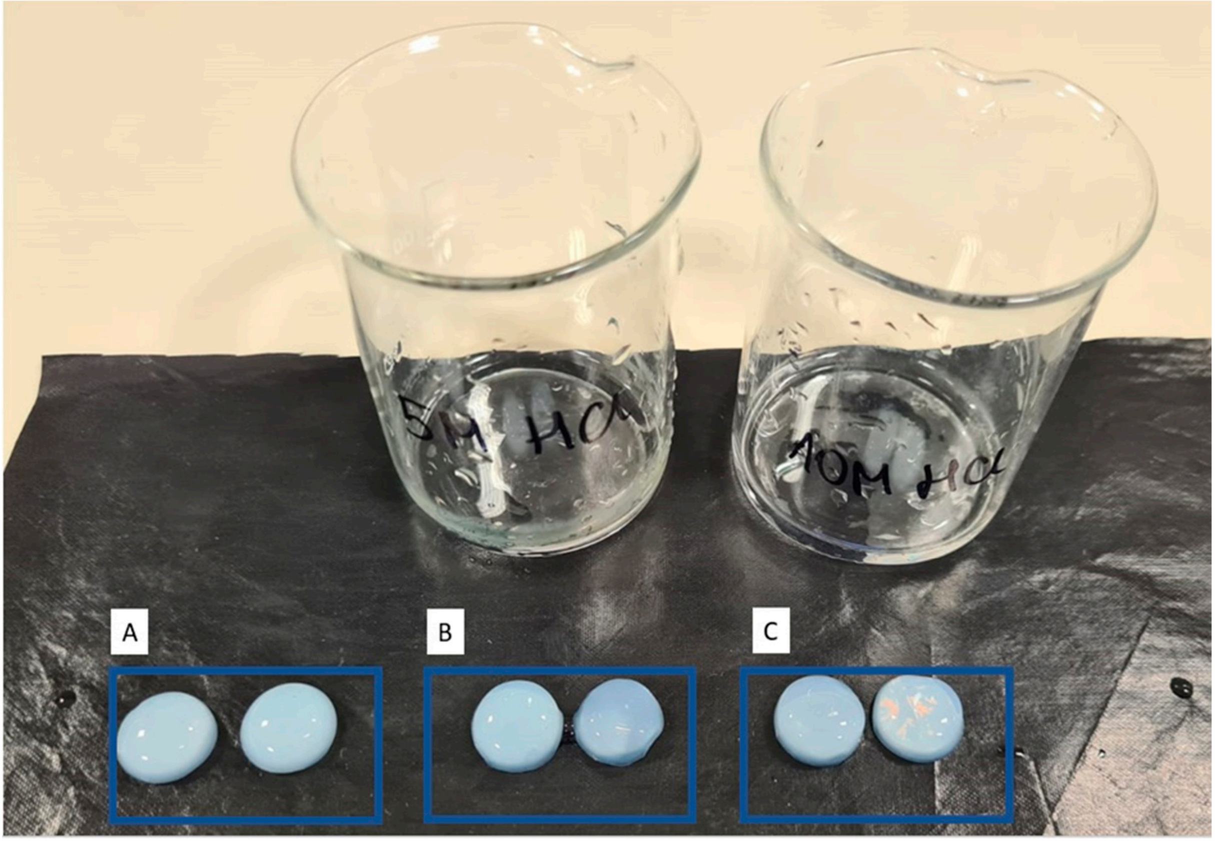

Figure S1. Bacterial cellulose (BC) incubated in low pH does not alter its structure. (A) control setting, BC incubated in saline for 4 h; (B) BC incubated in 5 M HCl for 4 h; (C) BC incubated in 10 M HCl for 4 h.

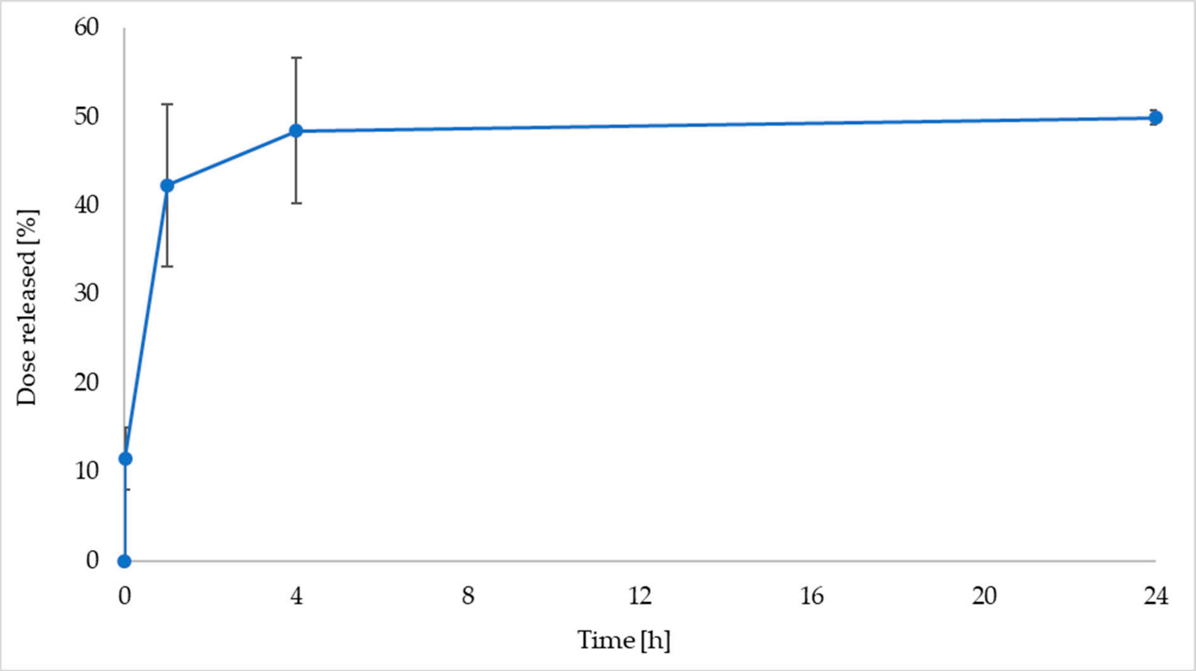

Figure S2. Time-dependent release of 3-bromopyruvate (3-BP) from bacterial cellulose (BC) disks.

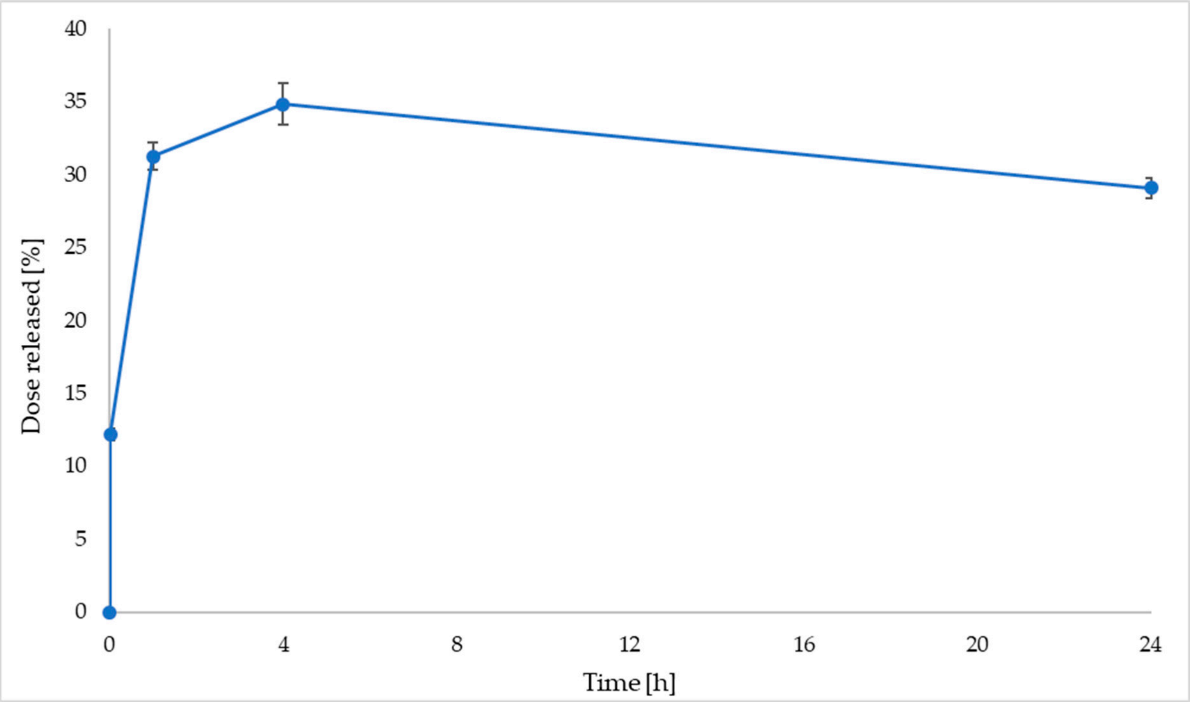

Figure S3. Time-dependent release of sertraline (SER) from bacterial cellulose (BC) disks.

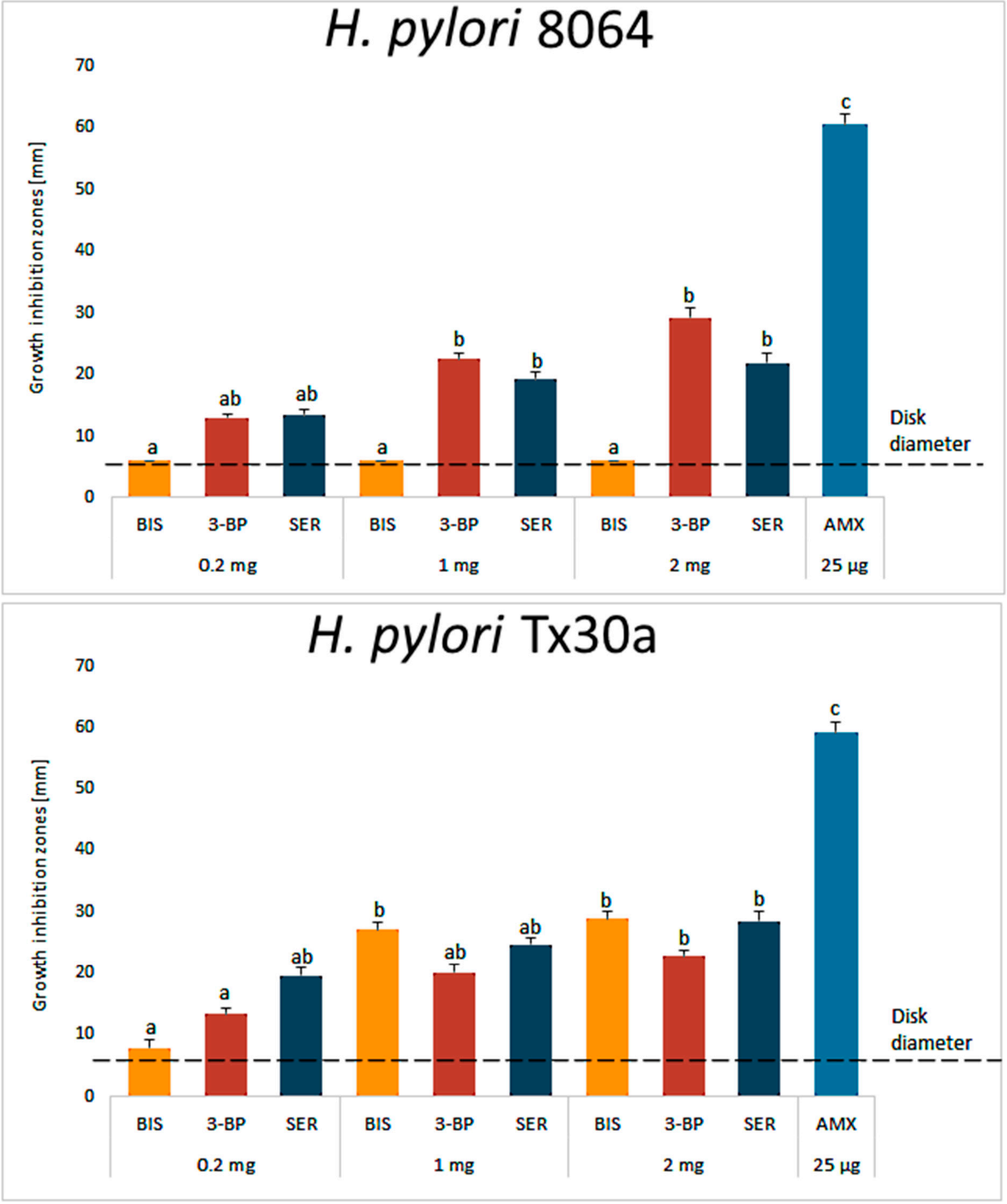

Figure S4. Activity of amoxicillin (AMX), bismuth subsalicylate (BIS), sertraline (SER), and 3-bromopyruvate (3-BP) released from paper disks against *H. pylori* 8064 and Tx30a strains measured by a modified disk-diffusion method. The dot-line at the value of 6 mm represents the diameter of paper disks. Columns with the same subscript letters are not significantly different from each other ($p > 0.05$).

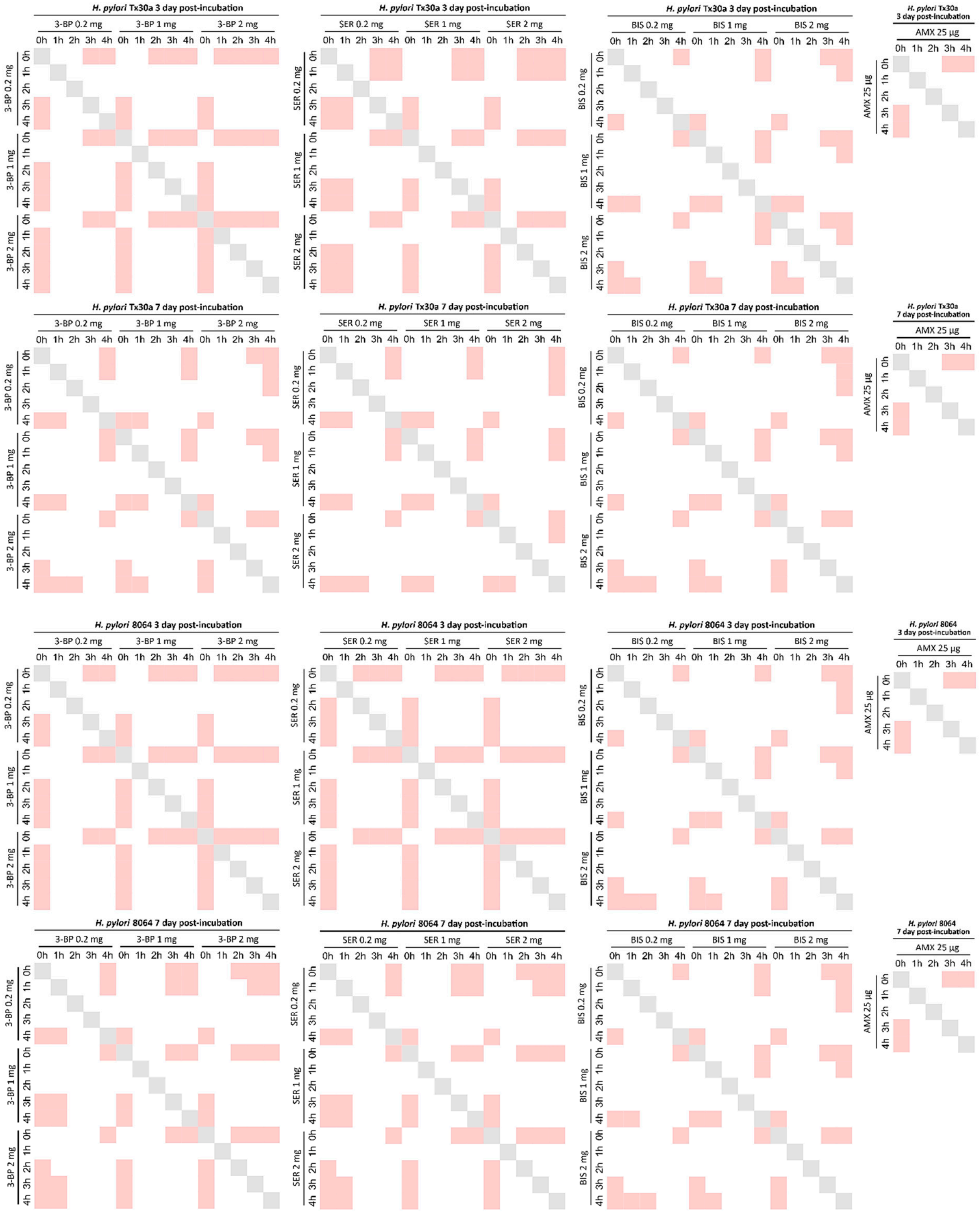

Figure S5. Checkerboards showing statistically significant differences between the viability of lawn biofilm cells of *H. pylori* Tx30a and 8064 strains exposed to BC carriers chemisorbed amoxicillin (AMX), bismuth subsalicylate (BIS), sertraline (SER), and 3-bromopyruvate (3-BP) over time. The red and white fields represent a statistically significant ($p < 0.05$) and not significant ($p > 0.05$) differences, respectively (K-W test with a post-hoc Dunn's analysis). Graphical presentation of the results made on the basis of Tripathy et al. (2020) [66].
